# Animal Reproduction journal and International Symposium on Animal Biology of Reproduction: Historical and personal reflections on two decades of exciting amalgamation of young and old science and scientists

**DOI:** 10.1590/1984-3143-AR2024-0078

**Published:** 2024-07-15

**Authors:** Luiz Renato de França

**Affiliations:** 1 Universidade Federal de Minas Gerais, Belo Horizonte, MG, Brasil

**Keywords:** Brazilian College of Animal Reproduction (CBRA), Animal Reproduction journal (AR), International Symposium on Animal Biology of Reproduction (ISABR), CBRA 50th anniversary, AR 20^th^ anniversary and ISABR 10^th^ edition

## Abstract

In 2024, the Brazilian College of Animal Reproduction (CBRA in Portuguese) is proudly celebrating its golden 50^th^ anniversary. Founded in 1974, CBRA has had a very productive and challenging journey of five decades, achieving many important milestones that have established it as a major society and its journal as a major reference in the field of animal reproduction, both in Brazil and internationally. Coincidentally, the *Animal Reproduction* journal and the International Symposium on Animal Biology (ISABR), both created and sponsored by CBRA, are also celebrating their 20^th^ and 10^th^ anniversary and edition, respectively, this year. These remarkable events are being celebrated in the city of Fortaleza, Brazil, during the 10^th^ edition of ISABR. As someone who had the privilege of playing a leading role in the creation and establishment of both *Animal Reproduction* journal and ISABR, I am honored to describe here the favorable circumstances that led to these significant achievements. The crucial steps and combined efforts required to make these institutions successful were unconditionally supported by the CBRA. Additionally, significant global networking and scientific collaborations, both individual and collective, have been pivotal in advancing the science and connecting the scientific community, spanning both young and experienced members, for decades. Finally, I hope that this historical article will inspire future generations of scientists in the field to continue CBRA’s journey and leadership, ensuring the growth of *Animal Reproduction* and ISABR advancement to even higher standards.

## Historical background and Animal Reproduction journal creation

The Brazilian College of Animal Reproduction (Colégio Brasileiro de Reprodução Animal – CBRA, in Portuguese) was founded in August, 1974, in the city of Belo Horizonte (state of Minas Gerais, Brazil), where it has remained since its foundation. Under the leadership of highly dedicated and idealistic professionals and professors, from both basic and applied field of reproduction, the mission of CBRA was committed to teaching, research, and the promotion of activities relevant to animal reproduction in all its branches. By the middle of 1980´s, when I was a graduate student in reproductive biology at the Federal University of Minas Gerais (UFMG), I started attending the excellent meetings organized by CBRA. Soon thereafter, having returned from my postdoc in the USA, I presented a talk at the XI Brazilian Congress on Animal Reproduction (Belo Horizonte, MG, October, 1995). Having gained a strong desire to help expand Brazil’s reach around the world, I organized an international session during this meeting and was able to invite three eminent scientists from the USA (Drs. Ruppert Amann, Lonnie Russell, and Larry Johnson). Thus, the international symposium embryo was implanted by the middle of 1990´s with CBRA sponsorship.

In the current year of 2024, CBRA is proudly celebrating its golden 50^th^ anniversary. During this very productive and challenging journey of five decades, probably one of the seminal CBRA periods was from 2003 to 2007, under the presidency of Dr. Rômulo Cerqueira Leite. In this special period, CBRA celebrated its 30^th^ anniversary and under the leadership of Drs. Luiz E.L. Pinheiro and Mark R.J.M. Henry (*in memoriam*) organized, for the first time in Latin America, the 15^th^ International Congress on Animal Reproduction (ICAR), a quadrennial and most consequential meeting in the field. This outstanding and traditional congress took place in the Convention Center of Porto Seguro city (Bahia state, Brazil, 8 – 12 August, 2004). I was surely very fortunate to have an active role in this meeting, participating, as one of the four members of the committee, in the evaluation of many hundreds of abstracts. Also, together with my thoughtful friend and colleague, Dr. Rex A. Hess, from the University of Illinois (USA), I coordinated a session in ICAR related to the “Germ cell transplantation and testis graft in mammals and fish”, which was, at that time, a novel and very exciting biotechnology approach.

**Figure 1:**
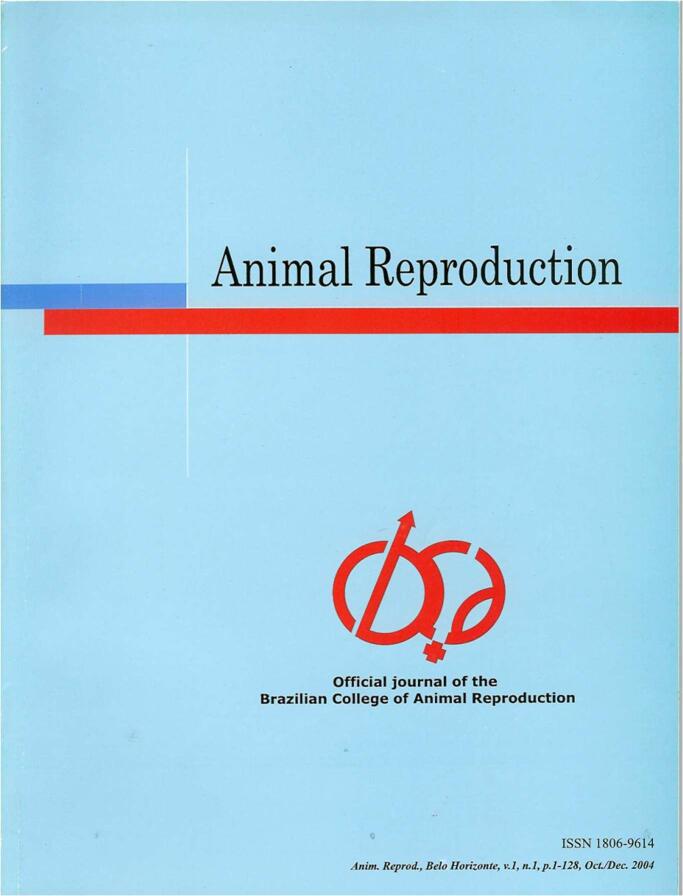
Inaugural issue cover from the of Animal Reproduction (AR) journal that was launched in the second semester of 2004.

With the special and fruitful momentum of CBRA, and the unique opportunity provided by the ICAR meeting organization, I had the honor and the privilege to be invited to take the leadership for the creation of a new and long-awaited international journal sponsored by CBRA. This very important task was accomplished together with Dr. Fernanda da Cruz Landim e Alvarenga (UNESP/Botucatu) and Dr. Eduardo Paulino da Costa (Federal University of Viçosa/UFV). During this process, we carefully discussed all the important scientific and technical aspects related to establishing this journal, such as the journal name “*Animal Reproduction”*, journal cover design, guidelines for authors, invitation criteria for international and Brazilians editorial board members. In this way, we could expect that *Animal Reproduction* would truly and significantly contribute to the field and become a standard high level international journal and, to our knowledge, the first scientific journal in Latin America to be published in English and exclusively devoted to the field of reproduction. After all these necessary procedures of divulgation and invitations for articles and reviews submissions, the inaugural issue of *Animal Reproduction* (Figure 1) was published in the second semester of 2004 (Please see Animal Reproduction, 2004). Specific information related to the editorial board of this first issue, as well as the important Editorial message from CBRA (see below), are found in the journal’s pdf link.

The Brazilian College of Animal Reproduction is proudly celebrating this year its 30^th^ anniversary and organizing the 15^th^ International Meeting on Animal Reproduction (ICAR), which for our pleasure is taking place in Brazil. We also have been publishing the Brazilian Journal of Animal Reproduction during the past 28 years. This conjunction of special events, and the awareness that we are reaching maturity, made us quite confident in pursuing new challenges and horizons. In this context we are now launching a new international journal in the field of Animal Reproduction, whose aims are to publish reviews and original articles related to the basic, applied and biotechnological aspects of animal reproductive biology. We are fully aware that this will be a difficult task. However, we are working very hard to make this a successful journal with the hope of fulfilling a longstanding need in Latin America by providing an excellent avenue for publications in this important and productive field of science. In this regard, we ask that you submit your next work for publication in our journal and we appreciate very much those scientists who kindly submitted their best research, which is now being published in the inaugural issue of Animal Reproduction.

Brazilian College of Animal Reproduction

Editors and Editorial Board of Animal Reproduction.

Looking back over our strategies for launching *Animal Reproduction,* its success is probably best measured by the fact that most of the original publications in the first issue have been well cited in the literature, as noted in JCR/Web of Science and Google Scholar. In fact, as of June 2024, many of these papers have been cited more than one-hundred times (please see the references list); for example, Sartori et al. (2004) (126 citations), Ginther et al. (2004) (118 citations), and Leal et al. (2004) (103 citations), which were studies involving respectively heifers, mares and goats, showing, as expected, the strength and the broad range of *Animal Reproduction* in relation to comparative reproductive biology, and species diversity. Also, the first review published in this inaugural issue (The role of estrogen in testis and the male reproductive tract: a review and species comparison, by Hess and Carnes (2004), is now cited almost 190 times. Taking into consideration the new era of *Animal Reproduction*, the most cited publication, Sheldon and Owens (2017), entitled “Postpartum uterine infection and endometritis in Dairy cattle”, has received 161 citations, which should be considered very good, according to the international standards, because the journal was not indexed by PubMed until 2017.

It just seemed natural that I would serve as journal Editor-in-Chief of *Animal Reproduction* from 2004 to 2015. My broad background in reproductive biology and love of science helped me through this relatively long period, but most importantly, I had invaluable help by several Co-Editors-in-Chief, which included Dr. Fernanda da Cruz Landim e Alvarenga (2004 to 2006), Dr. Eduardo Paulino da Costa (2004 to 2006), Dr. Mário Binelli (2005 to 2013), and Dr. José Ricardo Figueiredo (2007 to 2013). Conveying also great leadership, enthusiasm and dedication, from 2007 to 2013 the position of Editor-in-Chief was shared with a good friend and colleague, Dr. Eduardo Leite Gastal, who was before the Co-Editors-in-Chief (2005 to 2007). Drs. Antônio de Pinho Marques Jr and Carlos Eduardo Ambrósio shared the position of Editor-in-Chief in 2015, and since this year Dr. Ambrósio has taken this honored task with great dedication and enthusiasm, leading the journal to higher standards and international scientific visibility in many important aspects. As of 2024, the Animal Reproduction editorial members are as follows: Editor-in-chief, Dr. Carlos Eduardo Ambrósio (FZEA/USP, SP, Brazil); Co-editors-in-Chief, Dr. Felipe Perecin (FZEA/USP, SP, Brazil) and Dr. Ivan Cunha Bustamante Filho (UNIVATES, RS, Brazil); Associated editors, Dr. Angela Maria Gonella Diaza (University of Florida, FL, USA), Dr. Joanna Maria Gonçalves de Souza Fabjan (FV/UFF, RJ, Brazil), and Dr. Zamira Gibb (University of Newcastle, NSW, Australia).

## Animal Reproduction journal: the thrilling saga for an impact factor

After the fairly well-planned strategies for the creation of *Animal Reproduction* and having the first issues being published, several challenges were naturally expected. Among them we could mention the continuation of submission of enough good quality scientific papers and reviews, from different countries and continents. Also, because we had decided that *Animal Reproduction* would be published quarterly, the journal needed regularity that could be provided in large extension by good international visibility. In particular because around forty published papers would be necessary in total (8-10 papers per issue), in order to fulfill the four annual issues. The combination of these important aspects would allow us to apply for the *Animal Reproduction* indexation in the Journal Citation Report (JCR)/Web Of Science (WOS), which would provide the journal its first impact factor (IF). A very important scientific journal metric that, according to JCR, represents “the average number of times articles from the journal published in the past two years have been cited in the JCR year; being calculated by dividing the number of times a journal is cited in the JCR year, by the number of articles published in the journal in the previous two or five years (Clarivate, 2021).”

In reality, like an uphill battle, the IF saga became much more difficult than expected, mainly because we did not get enough manuscripts to be regularly published in the years 2007, 2008 and 2011. In this regard, we clearly realized that obtaining good quality submissions would be a difficult task without a published Impact Factor, creating a chicken-and-egg dilemma. However, all of our efforts were finally rewarded and we were able to cross the JCR tricky river, having the first *Animal Reproduction* IF of ~0.8 in 2015, which could be considered acceptable for a young Latin America journal. Since 2015, like a virtuous cycle, and in great part due to the indexation by PubMed in 2017, the IF increased gradually reaching almost 2.0. In the most recent evaluation, i.e. 2023/2022, the IF was 1.7, and we could expect a little increase for the upcoming 2004/2023 calculation.

It is probably worth give some perspective to the *Animal Reproduction* IF and other journal metrics. According to JCR/WOS in 2023 there were about 21,500 journals that encompassed about 250 disciplines from 112 countries, with almost two-thirds of these being scientific journals (ABCD, 2023). It is not surprising that only 2% from these journals were Brazilian-indexed publications. But what is surprising is that of these more than four-hundred Brazilian journals, *Animal Reproduction* is number 43. Thus, our journal is among the top ten percent of the Brazilian journals that had the best IF. Considering only the veterinarian-indexed journals in Brazil, *Animal Reproduction* is # 2 and worldwide in this specific field, its position is close to # 100. For comparative purposes, a publication can be evaluated also by its different subareas, which is expressed in quartiles (Q). According to SCImago Journal Rank (SJR), the best quartile for the Journal is Q2 in the field of “Animal Science and Zoology”. Indicating that in this particular area *Animal Reproduction* is within the 25 to 50% publications, which represents the second-best group. The Brazilian federal agency CAPES (Coordination for the Improvement of Higher Education Personnel) is a unique institution currently in charge of almost 370,000 graduate students, within almost 7,000 Graduate Studies Program (*stricto sensu*). CAPES has also a very important ranking system (CAPES/Qualis) for the evaluation of Brazilians and international publications/periodicals, according to their international relevance, comparatively and in their respective areas. Based on the CAPES/Qualis criteria used, *Animal Reproduction* is currently ranked A2 category, which is quite good, giving it a good reputation and visibility within the scientific community. Taken together, all the evaluations considered above indicate that this special Journal is a relevant scientific Brazilian publication, having also a reasonably good international impact in the field, as described below in more details.

One of the very useful parameters by which to evaluate a journal’s international relevance is the distribution of authors affiliation by country. This important evaluation is found in the JCR/SciELO (Scientific Electronic Library Online) database. Therefore, considering a universe of almost four hundred author affiliations it can be observed that, since it was indexed in JCR/WOS, *Animal Reproduction* had publications originating from almost fifty different countries, representing all the continents on our planet, as follows: nine (20%) from the Americas (North and South America); eighteen (39%) from Europe; fourteen (30%) from Asia; four (9%) from Africa; and one (2%) from Oceania. Regarding the number of authors, as expected, 45% were from Brazil, whereas both USA and China had 6%. These rather positive observations above clearly demonstrated that *Animal Reproduction* reached its initial goals, becoming a solid and internationally well-recognized scientific publication.

In another important parameter, since when it was created in 2004, almost two thousand manuscripts have been submitted to *Animal Reproduction*. To date, approximately 58% of them were published and 40-45% rejected, which is expected and within the range for most scientific journals with similar standards of quality. The time elapsed between the submission and acceptance/publication of an article is also an important factor to be considered for a scientific journal, and around 130 days are necessary to accomplish this task for the Journal. As of May 2024, there have been 75 issues of *Animal Reproduction* published, which I must say has taken diligent collective effort.

Finally, the criteria used for indexation of a journal follows strict rules; therefore, the evaluation of the indexes where a publication is included is an excellent indicator of its scientific quality. In this Regard, thanks to the continued support from CBRA and extensive efforts from the editors, *Animal Reproduction* is now indexed in the most important and currently available databases, as follows: Biological Abstracts; Biosis Previews; CABI Abstracts; CAPES/Qualis; Current Contents: Agric Biol Environ Sci; EBSCO; Directory of Open Access Journals; Google Scholar; PubMed Central; Scielo Brasil; Scopus/SJR; and Web of Science JCR/ISI. Also, in both its online (ISSN 1984-3143) and print (ISSN 1806-9614) versions, Animal Reproduction is sponsored by the two main Brazilians research institutions: CAPES and CNPq (National Council for Scientific and Technological Development).

## International Symposium on Animal Biology of Reproduction and Animal Reproduction journal: almost twin brothers

During the remarkable year of 2004, in which the ICAR meeting occurred and the Animal Reproduction journal was launched, we started discussions about the creation of an international symposium sponsored by CBRA. Interestingly, the spark that set this idea in motion first occurred in Dunblane, Scotland, during the 13^th^ European Workshop on Molecular and Cellular Endocrinology of the Testis, also in 2004. As I was already a regular participant of this traditional series of meetings, and therefore well integrated with the European scientific community in this field, I was invited to chair a morning session on the role of estrogens on male reproduction. Before this session started, as a joke I cheerfully announced that the next European Testis Workshop (in 2006) would be taking place in Rio de Janeiro, Brazil. Because it was so cold in Scotland, the participants joyfully and loudly celebrated this announcement. To my surprise, Dr. Brian Setchell (*in memoriam*) took my joke seriously and during the coffee-break came to me asking for more details about the meeting in Rio de Janeiro. I quickly changed the subject and asked him if he had ever visited Brazil, he replied saying that he had never been in Brazil and even in South America. I asked him why and he said that he was never invited or had the opportunity to do so. To put that in context, Dr. Setchell, who is still considered one of the finest andrologists ever worldwide, was very famous for his seminal studies in testis physiology and male reproduction, in particular for the pioneering research on the effects of heat on spermatogenesis and sperm function. His publications are truly masterpieces. Moreover, due to his broad intellectual and artistic skills he was also known as “A true Renaissance man”, being at that time a Professor Emeritus and Honorary Visiting Research Fellow at the University of Adelaide. Thus, at that particular moment in Dunblane, Scotland, I quickly planned in my mind that it was the right time to start an international meeting in Brazil in the field of reproduction, having Dr. Brian Setchell as the honored keynote speaker.

It is also important to mention that, at the Institute of Animal Physiology (Babraham/University of Cambridge, England), in 1974 and 1975 Dr. Setchell was the postdoc supervisor of Dr. Hugo Pereira Godinho, who was from the Federal University of Minas Gerais (UFMG/Brazil). After coming back to Brazil, Dr. Godinho fully implemented, at the Institute of Biological Sciences (ICB/UFMG), the research related to the testis and spermatogenesis in mammals and fish, the research I was very fortunate to continue and even expand during my entire scientific career at UFMG, after Dr. Godinho retirement. Therefore, I could be proudly considered Dr. Setchell´s great-grandson.

The brainstorm in Scotland regarding the creation of an international symposium in Brazil continued passionately and more vigorously in 2005, with strong support of the CBRA. In this regard, together with Dr. Hugo Pereira Godinho, and based on my previously successful strategies used for the creation of *Animal Reproduction*, all the details about this symposium were carefully discussed. At the beginning, we decided that the meeting name would be International Symposium on Animal Biology of Reproduction, which would give the nice acronym ISABR, being BR at the end of the acronym a hint for Brazil. Also, it was established that the symposium would encompass both male and female and all vertebrate species, covering therefore a wide-range of basic and biotechnological subjects or areas in reproductive biology. In addition, to stimulate the practice or even to improve the students and professionals proficiency, the entire symposium would be exclusively in English, including all the symposium information and guidelines for abstract submissions. This could also give to the students, post-docs and professionals, the opportunity to present their work as oral presentations.

CBRA has had since its foundation a traditional biennially national meeting (Brazilian Congress on Animal Reproduction), with a main focus on applied animal reproduction. Therefore, we decided that ISABR would occur also biennially, but in even years and intercalated with the other national meeting organized by CBRA that takes place in odd years. In this way, CBRA could organize a meeting every year for different audiences, being one meeting in Portuguese and the other one in English.

**Figure 2:**
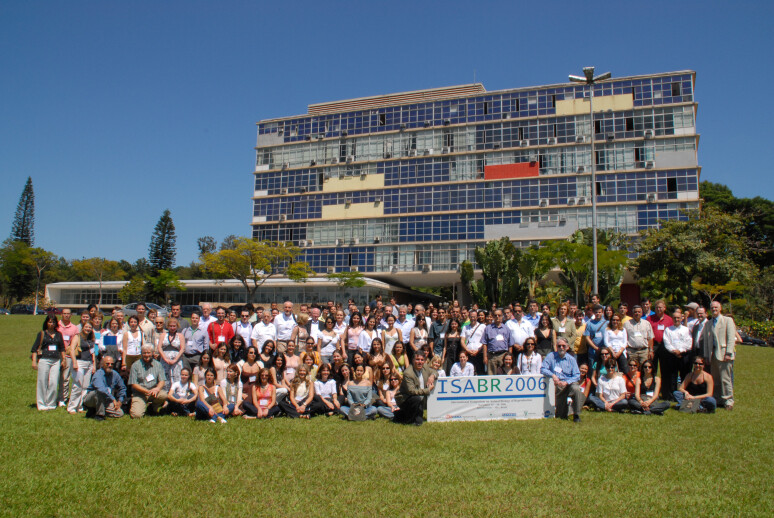
Participants of the first edition of the International Symposium on Animal Biology of Reproduction (ISABR), which took place at the Federal University of Minas Gerais (UFMG) campus, in November 15-18, 2006.

**Figure 3:**
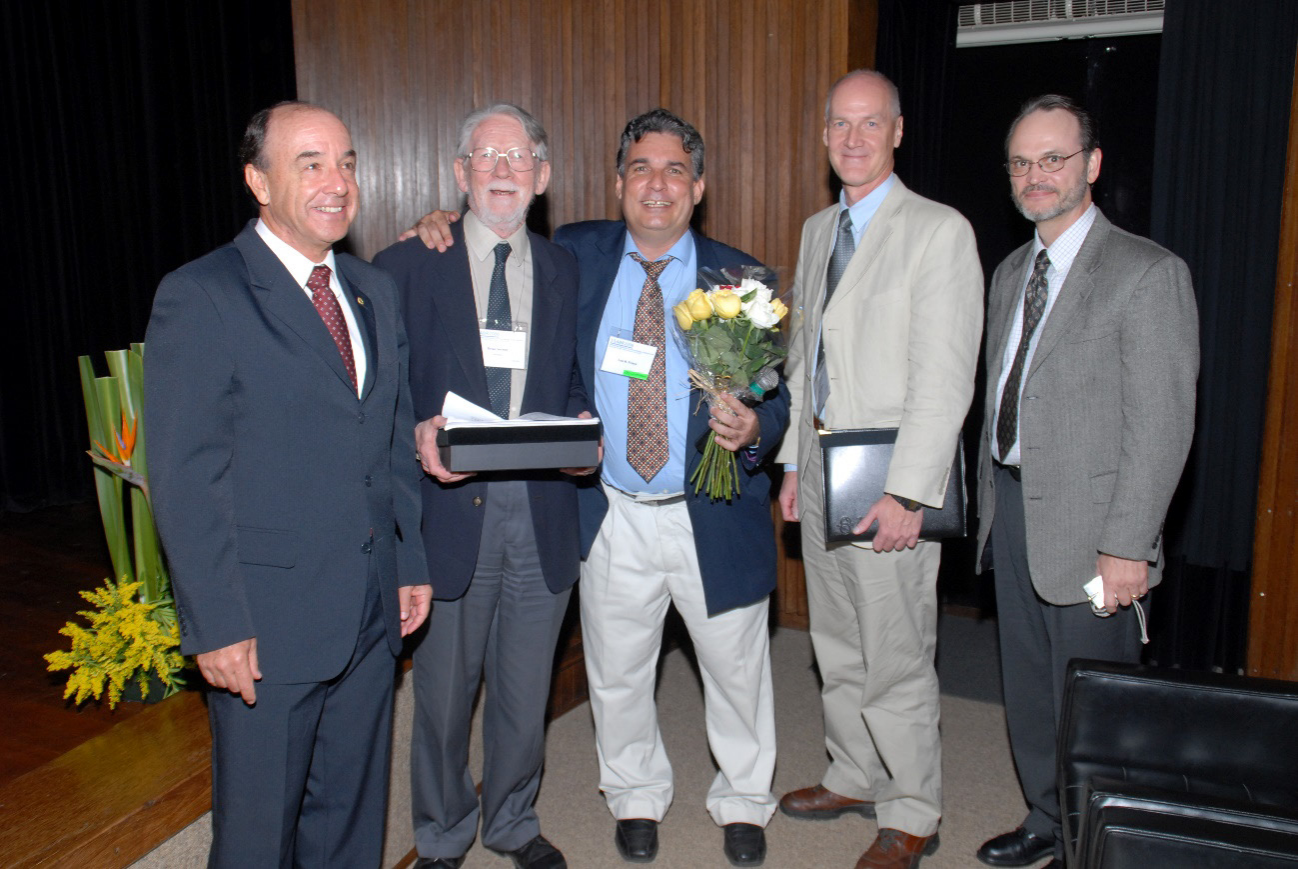
Opening session of the first edition of the International Symposium on Animal Biology of Reproduction (ISABR). From left to right: Dr. Carlos Alberto P. Tavares, Dean of Research of the Federal University of Minas Gerais (UFMG); Dr. Brian P. Setchell (University of Adelaide, Australia), Honored Professor and also the Keynote Speaker; Dr. Luiz Renato de França (UFMG), ISABR Chair; Dr. Barry T. Hinton (University of Virginia, USA), former Dr. Setchell´s PhD student; and Dr. Rex A. Hess (University of Illinois, USA), ISABR Secretary and Ceremonialist. Dr. Setchell´s 75^th^ birthday was also celebrated in this session.

After all the necessaries discussions and for logistical reasons, such as financial costs, meeting venue, divulgation, establishment of a scientific committee, sources of support and funding, dates for abstract submission, speakers’ invitations, among others, we decided that the ISABR first edition would occur by the middle of November, 2006. The venue chosen was the very fine campus of the Federal University of Minas Gerais (located in the city of Belo Horizonte, MG), in which all the required facilities and restaurants were available. Also, in its original format the meeting would last four days and, importantly, all meetings would have a keynote lecture delivered by the honored speaker, who should be preferentially a retired highly internationally recognized scientist, but still scientifically active. Following these considerations, the title chosen for the first meeting edition was “International Symposium on Animal Biology of Reproduction: From sex differentiation to reproductive biotechnology” (Figure 2), and the honored professor was Dr. Brian P. Setchell, who presented the talk “The effects of heat on the testes of mammals”. By a very fortunate coincidence, during the opening ceremony we had the opportunity to celebrate in great style his 75^th^ birthday (Figure 3).

Taking the fortuitous convenience that the Animal Reproduction journal was already a well-established publication, all the submitted abstracts, reviews, original articles and information related to the first ISABR were published in a special journal issue (please see França and Godinho, 2006), in which the historical Editorial message (see below) was also available.

This special issue of Animal Reproduction is a product of an exciting and unique conference–- The International Symposium on Animal Biology of Reproduction (ISABR): from Sex Differentiation to Reproductive Biotechnology, held in Belo Horizonte, MG, Brazil, November 15-18, 2006.The Federal University of Minas Gerais is honored to host the ISABR 2006, as this is an extraordinary opportunity to pay tribute to Dr. Brian P. Setchell and celebrate his 75^th^ birthday. This meeting covers very important and updated topics in reproductive biology, from fish to mammals and diverse areas of research and biotechnology. The inclusion of such a wide range of topics in reproduction is most appropriate for honoring Brian Setchell, as his research and teaching has had tremendous, wide-ranging influence on our current understanding of the male genital system, particularly testicular physiology. Many of his former students have continued as leaders in this field and his work has influenced nearly everyone who has trained in male reproductive biology over the past forty years. The timeless scientific journey of Brian Setchell, starting as Veterinary Research Officer in Australia at the Veterinary Research Station, Glenfield, New South Wales, has taken him to many countries and led to numerous positions of leadership and awards, as he has continued to pursue the advancement of scientific knowledge. He is currently Professor Emeritus and Honorary Visiting Research Fellow at the University of Adelaide. His dedication to quality science, and sustained education and leadership, even during his retirement years, serves as a model for all scientists and young trainees, as his commitment inspires a desire to learn and to reach for goals of distinction. Brian Setchell has been called “A true Renaissance man,” by many who know him. The ISABR 2006 meeting includes topics such as sex determination, testicular dysgenesis, spermatogonial stem cell biology, germ cell transplantation, germ cells gene regulation and expression, Sertoli cells hormonal regulation, sperm function, reproductive tract regulation, male contraception, toxicology, immunology, aging, and reproductive biotechnology. It is a great place to exchange knowledge and to be aware of advancements in reproductive biology. Scientists from many different countries and their Brazilian peers met in a unique forum promoting discussion on current scientific knowledge across numerous animal groups. Over twenty lectures were given by invited speakers and 150 posters presented.

The Federal University of Minas Gerais (http://www.ufmg.br) is a young university, but one with past and present scientists dedicated to the study of animal reproduction. It shares a vivid interest in reproductive biology and recognizes the importance of Setchell’s outstanding collaborations for her current achievements. Readers are invited to perusal this special issue of Animal Reproduction, which we believe provides a historical reference for many years.

Dr. Luiz Renato de França and Dr. Hugo P. Godinho

Organizing and Scientific Committee

Dr. Luiz R. França–- President (UFMG/CBRA) (França and Godinho, 2006, p. 79).

Due to the great scientific and cultural environment provided, for many years to come the first ISABR was considered a great success. Besides the 24 highly qualified speakers, from almost 10 different countries and 3 continents (North and South America, Europe, and Oceania), the first ISABR was attended by 250 participants and more than 150 abstract were presented as posters. Please see the complete program in the pdf link (Animal Reproduction, 2006). This meeting was also very well-funded by several agencies (CNPq, CAPES, and FAPEMIG), as well as by the Veterinary Regional Council (CRMV-MG). Once more, my good friend and research collaborator, Dr. Rex A. Hess - with whom I have now almost 20 scientific publications and have worked closely in the organization of many scientific meetings - was very generous and helpful. In this regard, besides being an ISABR speaker, he was kindly my personal and very dedicated secretary and the meeting ceremonial for the opening session. Also, as he is an excellent photographer and enjoy it very much as a hobby, he took hundreds of nice pictures during all the meeting activities and prepared many DVDs with these pictures, together with Dr. Setchells´s main papers PDFs. These DVDs Were graciously given as a gift to the participants at the end of the meeting. Symbolically, the first one to receive it was the honored and proudly happy speaker, Dr. Brian Setchell.

Besides the highly positive comments received for many and many years, the meeting scientific quality success could also be measured by the number of citations from the original publications and reviews published in the special issue of Animal Reproduction. For instance, as of June 2024, in JCR/Web of Science and Google Scholar, the review published by our honored speaker “The effects of heat on the testes of mammals, Setchell (2006)” is already cited 150 times, whereas the original article by Lacerda et al. (2006) “Germ cells transplantation in fish: the Nile-tilapia model” has 110 citations. Therefore, we believe that we accomplished our honorable task and that the first and historical ISABR edition paved, in the best way possible, the avenue for the subsequent meeting editions. In fact, the following ISABR meeting that occurred in 2008 at the University of São Paulo (USP, SP, Brazil) had 323 participants and 163 approved abstracts, including many from different countries. Due to a green card issue in the USA, at the last moment Dr. Gastal, who had personally and enthusiastically invited almost all speakers, could not come to Brazil to chair this excellent meeting. In this regard, given this unforeseen situation, I had the honor and the privilege to chair the second ISABR edition. In overall, including the current 10^th^ ISABR that is taking place in Fortaleza city in 2024, the estimated mean numbers of participants and abstracts for the ten editions are, respectively, around 250 and 170.

The information below is related to all ten ISABR Editions that occurred from 2006 to 2024, such as: date; city/state in Brazil where they took place; the meeting president/chair; the meeting main topic/focus; the honored/keynote speaker; the keynote lecture title; and the approximate numbers of participants (P) and abstracts (A) presented:

I/2006, Nov. 15-18 – Belo Horizonte/MG; Dr. Luiz Renato de França (UFMG, Brazil); *“From Sex Differentiation to Reproductive Biotechnology”*; Dr. Brian P. Setchell (University of Adelaide, Australia); *“The effects of heat on the testes of mammals”*; 250P and 175A.
II/2008, Nov. 18-22 – São Paulo/SP; Dr. Eduardo Leite Gastal (University of Wisconsin, USA) and Dr. Luiz Renato de França (UFMG, Brazil); *“Ovary: Folliculogenesis, Ovulation, and Luteolysis”*; Dr. Ronald H.F. Hunter (University of Cambridge, United Kingdom); *“Temperature gradients in female reproductive tissues and their potential significance”*; 325P and 165A.
III/2010, Oct. 22-24 – Águas de São Pedro/SP; Dr. Rodrigo Costa Mattos (UFRS, Brazil) and Dr. Mário Binelli (USP, Brazil); *“Epigenetic Control of Reproductive Processes”*; Dr. Bruce D. Murphy (Université de Montréal, Canada); *“Orphan nuclear receptor regulation of reproduction”*; 240P and 135A.
IV/2012, Oct. 17-20 – Campinas/SP; Dr. Arlindo A.A. Moura (UFC, Brazil); *“Environment and Reproduction”*; Dr. Gary J. Killian (USDA and The Pennsylvania State University, USA); *“Fertility-associated proteins in male and female reproductive fluids”*; 300P and 210A.
V/2014, Oct. 08-11 – Campinas/SP; Dr. Luiz Renato de França (UFMG, Brazil); *“Fertility: From Gametogenesis to Embryo Development”*; Dr. Rex A. Hess (University of Illinois, USA); *“Small tubules, surprising discoveries: from efferent ductules in the turkey to the discovery that estrogen receptor alpha is essential for fertility in the male”*; 250P and 155A.
VI/2016, Nov. 6-9 – Campos do Jordão/SP; Dr. Carlos Eduardo Ambrósio (USP, Brazil); *“Gametes versus Uterine Environment”*; Dr. Fuller Bazer (Texas A&M University, USA); *“Pregnancy Recognition Signals in Mammals: The Roles of Interferons and Estrogens”*; 370P and 230A.
VII/2018, Nov. 6-9 – Aracaju/SE; Dr. Carlos Eduardo Ambrósio (USP, Brazil); *“Reproductive Biotechnology and Future”*; Dr. Anthony M. Carter (University of Southern Denmark, Denmark); *“Evolution of placentation in cattle and antelopes”*; 170P 145A.
VIII/2021, Oct. 19-22 – Due to the Covid-19 pandemic, the meeting was online and occurred together with the “XXIV Brazilian Congress on Animal Reproduction”; Dr. Marcelo Rezende Luz (UFMG, Brazil); Dr. Eckhard Wolf (Ludwig Maximilians Universität, Germany); *“Advances in Animal Models and Reproductive Biology”*; 530P and 265A (joint meeting).
IX/2022, Nov. 23-25 – Bento Gonçalves/RS; Dr. Ivan Cunha Bustamante Filho (UNIVATES, Brazil); *“Trends in Molecular Regulation of Reproductive Processes”*; Dr. Graeme Martin (University of Western Australia, Australia); *“The 3 Ps of Reproduction (Pheromones, Photons, and Phood): a Reflexion on my Lifetime and Career in Science”*; 120P and 70A.
X/2024, Jul. 1-2 – Fortaleza/CE; Dr. Ivan Cunha Bustamante Filho (UNIVATES, Brazil); “New Frontiers in the Female Reproductive Biology”; Dr. Milo C. Wiltbank (University of Wisconsin, USA); *“Rethinking Programs in Reproduction to Optimize Gains in Reproduction and Management”*; 320P and 220A.

As it can be observed, in the ten ISABR editions herein listed, the honored keynote speakers are from prestigious institutions and universities from six different countries (USA, 4; Australia 2; and 1 from Canada, United Kingdom, Denmark, and Germany); and three different continents (North America, Europe, and Oceania). Another relevant aspect to be noted are the broad and scientifically vibrating focus chosen for each event, as well as the keynote very attractive subject titles, making them quite appealing for the eager audience and participants. Finally, demonstrating the outstanding international environment provided by the ISABRs, from the approximately 160 lectures presented 75% were from foreigner scientists, mostly from the USA, being several of them from other South America countries (Uruguay, Chile, and Argentina). This clearly indicates the CBRA leadership in South America, in the reproductive biology field.

## The universe of science: DNA expression, epigenetics and beyond

As it occurs both in the universe and life, everything is connected in the scientific world. I will make a point and explain that statement more contextually below. In order to be a good scientist, many prerequisites, such as intelligence, adequate scientific background, formal titles, dedication, determination, and funding, among others, are required. Moreover, from the moment a future good scientist enters the university as an undergraduate student, it takes usually at least one decade to start receiving significant funding as a principal investigator, supervising graduate students, and publishing independently in good journals as a corresponding author. Also, as modern science becomes more complex and progress speeds up, much more elaborate and integrated knowledge, expensive tools, and substantially far more sophisticated facilities are needed to stay current; demanding therefore other individual qualities from scientists. In this way, networking and collaborative studies, for instance, are nowadays an essential component of science. How could one accomplish all these very complex conditions? Based on my long and expansive experience, I will try to give a personal interpretation on that, which hopefully will also show a link to the creation of the Animal Reproduction journal and the ISABR.

To begin, it was fortuitous that I sought out Dr. Lonnie D. Russell (*in memoriam*) as my postdoctoral supervisor in the early 1990´s. At the time, he was located in the Department of Physiology at Southern Illinois University (SIU) Medical School in the USA and was in the prime of his career, having shown himself to be one of the best scientists in my research field. I was quite familiar with his excellent and impressive number of scientific publications, but I had never met him personally and actually had never traveled out of Brazil. Therefore, Dr. Hugo Godinho, from my department at UFMG, helped me write a nice letter to Dr. Russell, asking him for a post-doc position, which to my great delight was successful. When I arrived at SIU I quickly realized that Lonnie Russell was a very charismatic and joking sarcastic person. Having these peculiar characteristics and being well-known in the field, we became immediate buddies and even brothers, and he rapidly introduced me to innumerable famous scientists in reproductive biology, from the USA and abroad, in the many meetings we attended together, such as those organized by the Society for the Study of Reproduction (SSR), American Society of Andrology (ASA) that also helps to organize the North American Testis Workshop, and American Society for Cell Biology (ASCB). We also travelled together to meetings in Canada, Argentina, and to develop scientific activities in Europe (France). I should also mention that this incredible scientific network started when Dr. Rex Hess, who later became one of my best American friends and also a great brother, visited SIU to present an intriguing talk about the effects of hypothyroidism, during testis development, on testis function and sperm production. At SIU I also had the great pleasure of being introduced to Dr. Brian Setchell (our first ISABR honored keynote speaker), from the University of Adelaide, who was invited to present a talk about his seminal work on the effects of heat on the testes. In addition, during the many meetings I attended in the USA I had the opportunity to meet most of the bright and young scientists from Brazil in the reproductive area, including for instance Drs. Maria Christina W. Avellar (UNIFESP/São Paulo), Wilma de Grava Kempinas (UNESP/Botucatu/São Paulo), and Arlindo A. Moura (UFC/Ceará).

In another relevant aspect, besides his great importance in publishing in scientific journals, around the period I was in the USA as a postdoc or doing work in collaboration, Dr. Russel published and edited many books that became groundbreaking references in the field, such as: “Histological and Histopathological Evaluation of the Testis (cited more than 2,550 times!)”; “The Sertoli Cell”; “The Leydig Cell”; “Electron Microscopy: Principles and Techniques for Biologists”; and “Molecular Biology Made Simple and Fun”. He was also the Editor-in-Chief of “Tissue and Cell” journal and, showing his already very high consideration and appreciation for Brazil, published in the year 2000 a special issue of the journal with Brazil drawn on the journal cover and having articles only from Brazilians authors, in celebration of 500 years of Brazil discovery. Sadly, and unexpectedly, Lonnie died in the year 2001 in Brazil, a country he loved so much, still very young at the age 57. In a more than deserved tribute to him, since the first edition of ISABR I have established the Lonnie Russell Lecture. A lecture that I was very happy to present in the VII ISABR, with the title “Sertoli cell: what can we learn from different vertebrate models?”, recognizing him for also being known in the scientific community as “Mister Sertoli”, due to his sincere devotion to this key cell that orchestrates spermatogenesis. The same devotion and appreciation I had for this amazing cell, even before I had met Lonnie Russell, during the initial steps of my graduate studies.

Even though I had tragically lost my great friend and collaborator, my global face-to-face networking continued to grow significantly and was now more directed to Europe, which led to an expansion into fish reproduction. Thus, I continued to develop scientific collaborations and to meet more and more eminent scientists and to attend the most important meetings on fish physiology of reproduction. Also, besides becoming a member of many scientific societies and being invited to join the editorial board of many scientifically relevant journals, I attended and organized meetings and symposium sessions and presented talks in countless number of meetings, research institutions and universities. At some point of my career, I had established collaborations with approximately 15 different countries, including Japan and our neighbor and soccer enemy Argentina, and co-authored papers with 20 to 30 prominent scientists from all five continents. In Brazil, I also had very fruitful collaborations and connections with many institutions and countless number of colleagues. This success is best reflected in the amazing number of participants (approximately 350) that attended in 2012 the first integrated symposium of graduate programs on cell biology from Minas Gerais state in Brazil, in which I was the honored professor and the keynote speaker. In this meeting, around 250 abstracts were presented and more than 20 close colleagues were speakers, including my good friends Dr. Rex Hess from the USA and Dr. Thierry Guillaudeux from France.

From 2014 to 2018 I had the great honor to become the director of the National Institute for Amazonian Research (INPA), which is considered the most important research institution in tropical biology in our planet. At the end of this very important period, I also had the privilege to organize and chair the 11^th^ International Symposium on Reproductive Physiology of Fish (ISRPF), which occurred in Brazil, in the city of Manaus, in 2018. The ISRPF is a quadrennial and most consequential meeting in the field and this particular edition occurred for the first time in Latin America. These personal events are mentioned because many of these network connections and collaborations resulted into papers and reviews that are now published in *Animal Reproduction*, and of special importance led to invited lectures to ISABR. In a virtuous cycle or win-win situation, these all created even more papers and review submissions to *Animal Reproduction*. I personally knew almost all of the invited speakers that presented lectures in the first ISABR edition, which is a testament to the great benefits resulting from efforts to establish a global network. Also, most of speakers were colleagues and collaborators, and even good friends. Surely, as a big global family, this is one of the most rewarding aspects of science.

Summarizing this topic, a DNA lineage that started fifty years ago in England, when Dr. Hugo Godinho from Brazil was supervised by Dr. Brian Setchell, was expressed in the USA almost twenty years later. Due to many positive epigenetic transformations, an amazing global network started and nicely expanded abroad, with crucial inputs from Dr. Lonnie Russell and Dr. Rex Hess, allowing at the same time the self-renew and differentiation of a germ stem cell. Importantly, with the generosity and favorable conspiracy of the universe, suddenly everything became aligned, and more DNAs were expressed in the very supportive and unselfish environment provided by the CBRA. Thus, in a kind of morphic resonance or morphogenetic field, these fruitful conjunctions resulted in the creation of [Bibr B002]*Animal Reproduction* journal and the International Symposium on Animal Biology of Reproduction. In this context, I am very proud that, along my scientific career and with the capacity to envision a better future for Brazil in my field of knowledge, I was able to collaborate for these very important CBRA achievements, keeping a strong sense of integrity and an unwavering commitment to ethical behavior. In this way, as a happily retired full Professor I believe that I have fully accomplished my personal goals. However, we still need to keep in mind that it is our obligation to help or find professionals/scientists that would be willing to put in the time and effort required to lead ISABR and the *Animal Reproduction* journal to even greater heights.

## Concluding remarks and future perspectives

According to Thomas Kuhn (Bird, 2018), science develops in leaps. That means progress does not occur simply due to the gradual accumulation of knowledge and/or experiments, but happens only after disruptions or the breaking of paradigms in so-called normal science. This has always occurred, as was seen during the great transformations in scientific models of the late 19^th^ and 20^th^ centuries. However, as described in quantum physics, this phenomenon of quantum leaps occurs only in the presence of the observer, and is reflected in the surrounding reality based on the person’s internal experiences; i.e. consciousness creates reality. When I was very young, I had a small zoo in my house. Naturally, I became a veterinarian student (at age 18) in 1974, in the same year CBRA was founded, and moved to Belo Horizonte in 1984 to start my graduate studies in reproductive biology. Therefore, I feel honored and privileged that CBRA allowed my childhood dreams to come true, leading to the creation and establishment of both *Animal Reproduction* journal and ISABR.

*Animal Reproduction* was born in 2004 at the right moment and in an appropriate and mature environment, in which CBRA was already a solid society in the field and celebrating its 30^th^ anniversary. Thus, the Journal was given good DNA that could be nicely expressed. The fact that CBRA was organizing at that time, and for the first time in Latin America, the ICAR meeting, was just a mirror of this quite fortunate moment. Besides that, the journal creation was spearheaded by well-established and internationally recognized scientists, who could envision a very nice future for such a publication. Moreover, they were quite determined and enthusiastically prepared to overcome the natural difficulties that appeared during the journey. Celebrating now in 2024 its 20^th^ anniversary, *Animal Reproduction* is proudly a solid Brazilian journal with international reach, having quite nice indicators of scientific quality. Given the favorable conditions provided by CBRA, in association with my already extensive global scientific network, in 2004 we also started discussions related to the creation of an international symposium, sponsored and supported by CBRA. These discussions progressed quite well and with great passion in 2005, leading in 2006 to the first biennial ISABR edition in the warm and receptive campus at the Federal University of Minas Gerais. This meeting was considered a great success in all possible aspects, highly stimulating the subsequent ISABR editions that were similarly very well attended and appreciated. In this regard, similar to *Animal Reproduction*, in a couple more editions, ISABR became a traditional meeting in Brazil and South America, becoming an anticipated meeting by the scientific community in this field. Importantly, as very close brothers, the *Animal Reproduction* and ISABR became very well integrated and a part of not only the Brazilian scientific community but also the international network. Thus, the now more experienced and grounded big father CBRA expanded its natural leadership to new scientific frontiers abroad, having therefore an excellent opportunity to develop bridges with other societies and institutions. As a final note, we expect and wish long life to the ISABR and to the Brazilian-based Journal, and that both CBRA scientific enterprises continue their successful odyssey. Moreover, we hope that they could be warmly embraced by enthusiastic young scientists and experienced leaders from the field. Just remembering that, "*Dare is possible, the future is now, and the best is yet to come*."
